# Small Extracellular Vesicles Secreted by iPSC-Derived MSCs Ameliorate Pulmonary Inflammation and Lung Injury Induced by Sepsis through Delivery of miR-125b-5p

**DOI:** 10.1155/2023/8987049

**Published:** 2023-07-01

**Authors:** Wei Peng, Yun Yang, Jiaquan Chen, Zeyao Xu, Yuanlei Lou, Qi Li, Ning Zhao, Kejian Qian, Fen Liu

**Affiliations:** ^1^Department of Critical Care Medicine, The First Affiliated Hospital of Nanchang University, Nanchang, Jiangxi, China; ^2^Medical Innovation Center, The First Affiliated Hospital of Nanchang University, Nanchang, Jiangxi, China; ^3^Department of Critical Care Medicine, The People's Hospital of Fengcheng City, Yichun, Jiangxi, China; ^4^Institute of Urology, The First Affiliated Hospital of Nanchang University, Nanchang, Jiangxi, China

## Abstract

**Background:**

Sepsis-induced acute lung injury is a common critical illness in intensive care units with no effective treatment is currently available. Small extracellular vesicles, secreted by mesenchymal stem cells (MSCs), derived from human-induced pluripotent stem cells (iMSC-sEV), possess striking advantages when incorporated MSCs and iPSCs, which are considered extremely promising cell-free therapeutic agents. However, no studies have yet been conducted to systemically examine the effects and underlying mechanisms of iMSC-sEV application on attenuated lung injury under sepsis conditions.

**Method:**

iMSC-sEV were intraperitoneally administered in a rat septic lung injury model induced by cecal ligation and puncture (CLP). The efficacy of iMSC-sEV was assessed by histology, immunohistochemistry, and pro-inflammatory cytokines of bronchoalveolar lavage fluid. We also evaluated the in vitro effects of iMSC-sEV on the activation of the inflammatory response in alveolar macrophages (AMs). Small RNA sequencing was utilized to detect changes in the miRNA expression profile in lipopolysaccharide (LPS)-treated AMs after iMSC-sEV administration. The effects of miR-125b-5p on the function of AMs were studied.

**Results:**

iMSC-sEV were able to attenuate pulmonary inflammation and lung injury following CLP-induced lung injury. iMSC-sEV were internalized by AMs and alleviated the release of inflammatory factors by inactivating the NF-*κ*B signaling pathway. Moreover, miR-125b-5p showed a fold-change in LPS-treated AMs after iMSC-sEV administration and was enriched in iMSC-sEV. Mechanistically, iMSC-sEV transmitted miR-125b-5p into LPS-treated AMs to target TRAF6.

**Conclusion:**

Our findings demonstrated that iMSC-sEV treatment protects against septic lung injury and exerts anti-inflammatory effects on AMs at least partially through miR-125b-5p, suggesting that iMSC-sEV may provide a novel cell-free strategy for the treatment of septic lung injury.

## 1. Introduction

Sepsis, a common critical illness with high mortality rates worldwide, is defined as a life-threatening organ dysfunction resulting from a dysregulated host response to infection [1, 2]. Among the injured organs, the lung is the first and most vulnerable organ to fail. Approximately 40% of acute lung injury incidence results from sepsis [3, 4]. Sepsis-induced acute lung injury is characterized by an overwhelming and rapid intrapulmonary inflammatory response. In response to the invasion of microorganisms, alveolar macrophages (AMs), the main immune cells residing in the lung tissue, play a critical role in the pathogenesis of acute lung injury by overactivation and releasing excessive pro-inflammatory cytokines, such as TNF-*α*, IL-6, and IL-1*β* [5]. At present, there is still a lack of specific treatment for sepsis-induced acute lung injury. The overall treatment strategies for septic lung injury remain primarily supportive such as lung-protective ventilation, supportive care, and extracorporeal membrane oxygenation, while the mortality rate remains high [6]. Therefore, it is necessary to develop new therapeutic approaches to effectively alleviate the inflammatory response of septic lung injury.

Mesenchymal stem cells (MSCs) are multipotent stromal cells derived from various tissues, including bone marrow, adipose tissue, and umbilical cord blood [7]. MSCs can interfere with different pathways of the immune response and possess high immunomodulatory faculty [8, 9]. In preclinical studies, MSCs therapy could alleviate inflammatory response, mitigate pulmonary edema, and most importantly, improve survival in cecal ligation and puncture (CLP) constructed animal models of sepsis [10, 11]. Additionally, a clinical trial revealed that a single intravenous injection of human bone marrow-derived MSCs was able to achieve a certain curative effect in nine patients with ARDS [12]. The accumulated evidence supports that MSCs exerting anti-inflammatory effects in immune-mediated diseases such as sepsis or acute respiratory distress syndrome by secreting a range of growth factors, chemokines, and extracellular vesicles (EVs) [13–15]. Despite their attractive therapeutic effects, the application of tissue-derived MSCs faces several hurdles, such as low viability of the transplanted cells, innate heterogenicity, and decreased therapeutic efficacy during expansion [16, 17]. Small extracellular vesicles (sEVs), including classical exosomes, represent natural nanosized lipid bilayer vesicles with a diameter of 30–150 nm, which is released by almost every cell type that participates in intercellular communication via the delivery of bioactive molecules [18]. In this regard, the utilization of sEVs secreted by MSCs has been considered an attractive therapeutic agent to overcome the potential risks that arise from MSCs in clinical application [19, 20].

Induced pluripotent stem cells (iPSCs), as an alternative source of pluripotent stem cells, can be generated from patient-specific adult somatic cells through transcriptional factor-induced reprograming. iPSCs can be induced to differentiate into MSCs (iMSCs), owing to the high proliferative and differentiation potentials [21]. Compared with other tissue-derived MSCs, iMSCs possess a superior advantage in cell proliferation, immunomodulation, generation of EVs, and being an inexhaustible source of MSCs, which makes iMSCs become an ideal cellular source for broad-scale preparation of sEVs [21–24]. Despite the broad prospects of utilizing sEVs derived from iMSCs [25], the role of iMSC-sEV in the context of sepsis-induced acute lung injury is not clear. Thus, we aim to investigate the effects of iMSC-sEV on sepsis-induced lung injury and further explore the underlying mechanism that may contribute to the therapeutic effects of iMSC-sEV in pulmonary inflammation, providing a new cell-free strategy for the treatment of septic lung injury.

## 2. Materials and Methods

### 2.1. Characterization of iMSCs

The surface antigens of iMSCs were determined by flow cytometry analysis. iMSCs were trypsinized and washed twice before resuspending in phosphate-buffered saline (PBS) containing 2% fetal bovine serum (FBS) and 1 mM ethylenediaminetetraacetic acid.

Then cells were incubated in PBS containing 3% bovine serum albumin for 30 min to block nonspecific antigen binding. For cell surface labeling, cells were next incubated with 5 *μ*L of antibodies recognizing characteristic MSC-specific surface markers, including phycoerythrin (PE)-conjugated anti-CD29, PE-conjugated anti-CD73, PE-conjugated anti-CD90, PE-conjugated anti-human leukocyte antigen (HLA)-DR, fluorescein isothiocyanate (FITC)-conjugated anti-CD45, FITC-conjugated anti-CD45, and FITC-conjugated anti-CD34. Cell surface antigens were analyzed using a BD FACSCanto Flow Cytometer.

### 2.2. Collection and Identification of iMSC-sEV

Upon reaching 80% confluency, the culture media from the iMSCs was replaced with MesenGro hMSC medium (Stem RD), and the cells were subsequently cultured for an additional 48 hr. For sEVs isolation, a conditional medium was centrifuged sequentially at 300 × *g* for 10 min and then at 2,000 × *g* for 10 min. To remove the cellular debris in the conditional medium, the supernatants were filtered through 0.22 *μ*m pore filters. Finally, sEVs were isolated by ultracentrifugation at 100,000 × *g* for 2 hr, and the pellet was subsequently washed with PBS and subjected to ultracentrifugation. The amount of protein concentration in sEVs was evaluated by a micro bicinchoninic acid (BCA) protein assay kit (Pierce), according to the manufacturer's recommended protocol. The morphology of sEV was observed by transmission electron microscope (TEM). Nanoparticle analysis was conducted to quantify the diameters of sEV using a NanoSight LM10 instrument (Nano Sight Limited, Amesbury, UK). Antibodies against the CD9 and CD63 proteins were used to analyze the expression of sEV markers.

### 2.3. Cell Culture

Induced pluripotent stem cells derived from mesenchymal stem cells (iMSCs) were provided by Prof Wang Yang (Shanghai Jiaotong University, China). Cells were harvested and expanded in 0.1% gelatin-coated dishes containing MSC medium. Cells were serially passaged every 5–7 days until they formed a uniform fibroblast morphology, which was then evaluated based on MSC phenotypic characteristics and differentiation potential.

The rat alveolar macrophage cell line NR8383 was purchased from the Cell Bank of the Chinese Academy of Sciences and cultured in HamF-12K culture medium (Wisent, Canada) containing 15% FBS at 37°C in a 5% CO_2_ incubator. The culture medium was refreshed every 2-3 days. After reaching 80% confluence, cells were treated with 50 ng/mL of lipopolysaccharide (LPS) for 24 hr.

### 2.4. Rat Septic Lung Injury Model and Treatment

All procedures and protocols were approved by the ethics committee of the Animal Experimental Research Center of Nanchang University. Adult male SD rats weighing approximately 100 g (4-5 weeks old) were used in this study. To construct the model of septic lung injury, rats were anesthetized by intraperitoneal injection of 50 mg/kg pentobarbital and underwent CLP for sepsis. Briefly, a median incision of 3-4 cm was created to the abdominal wall, and the cecum and ileocecal mesentery were identified, followed by ligation at the site one-fourth of the distance upstream of the cecal end. An 18-gauge needle was used to puncture through the ligation site twice. The cecum was squeezed gently to extrude a small amount of fecal contents from the punctured cecum and transferred it to the peritoneal cavity; closed the incision wound was layer-by-layer, and administered lactated ringer solution (20 mL/kg) subcutaneously after the operation. Finally, the exposed abdominal incision was closed using two layers of running 4-0 silk sutures. In sham-operated rats, only laparotomy was performed, but their cecum was not ligated or punctured.

### 2.5. In Vitro iMSC-sEV Uptake Assay

To verify whether the iMSC-sEV were internalized into the AMs, iMSC-sEV were labeled with a PKH-67 green fluorescent cell linker kit according to the manufacturer's instructions. PKH-67-labeled iMSC-sEV were cocultured with AMs (NR8383) for 12 hr, and the nuclei were stained with 4′,6-diamidino-2-phenylindole. NR8383 cells were fixed, washed, and viewed with a confocal laser scanning microscope (Olympus).

### 2.6. Enzyme-Linked Immunosorbent Assay

The levels of TNF-*α*, IL-1*β*, and IL-6 were determined by ELISA kits (BOSTER Biosciences) according to the manufacturer's instructions. Standard curves were plotted to calculate the concentrations of the inflammatory factors in the samples.

### 2.7. Real-Time Quantitative PCR (qPCR)

The total RNA of the cells was isolated using an RNA extraction kit (TAKARA, Japan). After that, 1 *μ*g of total RNA was used for the cDNA synthesis with a PrimeScript reagent Kit (TAKARA, Japan). The polymerase chain reaction (PCR) reaction was performed in 20 *μ*L of the final volume, containing 0.08 *μ*M of forward and reverse primer, 2 *μ*L of 10–20× diluted original cDNA, and 10 *μ*L SYBR Green PCR Master Mix using an Applied Biosystems 7,500 real-time PCR machine. After the reaction was completed, the amplification curve and the dissolution curve were confirmed. All results were calculated using the 2^−*ΔΔ*Ct^ method through normalizing to *β*-actin or U6. The miRNA qPCR primers were purchased from Guangzhou Ribobio Corporation.

### 2.8. Western Blot

Total protein was extracted for the western blot analysis using a RIPA lysis buffer (Beyotime Biotechnology, Shanghai) containing a protease inhibitor cocktail for 30 min. The protein was quantified by a BCA kit (Beyotime Biotechnology, China) and boiled at 95°C for 5 min. The protein suspension was separated into sodium dodecyl sulfate–polyacrylamide gel electrophoresis (PAGE) gels. Proteins were separated and electrophoretically transferred onto a polyvinylidene fluoride membrane using an electroblotting apparatus (Bio-Rad, USA). The membrane was incubated in 5% bovine serum albumin for 60 min at room temperature, followed by incubation overnight at 4°C with the primary antibody against CD9 (1 : 1,000), CD63 (1 : 1,000), p-p65 (1 : 1,000), p65 (1 : 1,000), glyceraldehyde-3-phosphate dehydrogenase (GAPDH) (1 : 10,000), *β*-actin (1 : 10,000), and TRAF6(1 : 1,000). The antibodies were purchased from Abcam. After washing, the blots were incubated with a secondary horseradish peroxidase-conjugated antibody for 1 hr at room temperature; the protein signals on the membrane were detected using a gel imaging scanning system (Bio-Rad, USA) and quantified by ImageJ (National Institutes of Health, USA) using *β*-actin or GAPDH as a reference protein.

### 2.9. Histopathology

Formalin-fixed and paraffin-embedded lung tissue blocks were sliced into 4 mm thick sections. For analysis of tissue inflammation, the lung sample sections were subsequently stained with hematoxylin & eosin and observed under a light microscope. Lung injury scores were estimated by Smith's scoring method, with a higher score indicating a more severe injury.

### 2.10. Immunohistochemistry

After deparaffinization and hydration through a graded series of alcohol to water, the lung tissue sections (4 mm) were incubated in 3% H_2_O_2_ and PBS to block endogenous peroxidase activity for 10 min. The sections were washed three times with PBS and incubated in normal goat serum for 15 min to block nonspecific binding. They were stained with anti-CD68 antibodies overnight at 4°C and then incubated with a goat anti-rabbit IgG antibody for 1 hr at room temperature. Peroxidase activity was visualized using a diaminobenzidine tetrahydrochloride solution (Cell Signaling Technology, USA). The sections were evaluated with an optical microscope (Olympus Optical, Tokyo, Japan).

### 2.11. Small RNA Sequencing

Total RNA was extracted from NR8383 cells using Trizol (Invitrogen, Carlsbad, CA, USA) according to the manual's instructions. The library was prepared with 1 *μ*g total RNA for each sample. Total RNA was purified by electrophoretic separation on a 15% urea denaturing PAGE gel, and small RNA regions corresponding to the 18–30 nt bands in the marker lane (14-30 ssRNA Ladder Marker, TAKARA) were excised and recovered. Then the 18–30 nt small RNAs were ligated to a 5′-adaptor and a 3′-adaptor. The adapter-ligated small RNAs were subsequently transcribed into cDNA by SuperScript II Reverse Transcriptase (Invitrogen, USA), and then, several rounds of PCR amplification with a PCR Primer Cocktail and PCR Mix were performed to enrich the cDNA fragments. The PCR products were selected by agarose gel electrophoresis with target fragments 100–120 bp and then purified by a QIAquick Gel Extraction Kit (QIAGEN, Valencia, CA). The library was qualitatively and quantitively assessed through two methods: the distribution of the fragment's size was checked using the Agilent 2100 bioanalyzer, and the library was quantified using real-time qPCR (TaqMan Probe). The final ligation PCR products were sequenced using the BGISEQ-500 platform (BGI-Shenzhen, China). The differential expression analysis was calculated between two sets of samples based on the significant criteria of |log_2_ (fold change) | ≥ 1 and *P*-value < 0.05. The target genes of the d miR-125b-5p were predicted by using TargetScan. Gene ontology (GO) and the Kyoto Encyclopedia of Genes and Genomes (KEGG) analysis of the predicted target genes were performed using DAVID Bioinformatics Resources 6.8.

### 2.12. Transfection

NR8383 cells were transfected with miR-125b-5p mimic (50 nM), miR-125b-5p mimic (50 nM), and control RNAs using Lipofectamine 2000 according to the manufacturer's instructions.

### 2.13. Plasmid Construction and Luciferase Assay

The 3ʹ-UTR segment of TRAF6 with the putative binding sites of miR-125b-5p was amplified by PCR using genomic DNA as a template. The PCR product was cloned into the psiCHECK-2 vector following the manufacturer's protocol (Promega) to obtain wild-type psi-CHECK-TRAF6-3ʹ-UTR (wt). Mutations (Mut-UTRs) were generated by a site-directed mutagenesis kit (Stratagene; Agilent Technologies, Inc., Santa Clara, CA, USA), yielding the Mut-UTR plasmid. A mutant of potential miR-125b-5p binding sites inWT-3′-UTR with a mutation (mut) of complementary sequences (termed psi-CHECK-2-TRAF6mut-3′-UTR) was generated using the QuikChange II Site-Directed Mutagenesis Kit (Stratagene, USA). Plasmid DNA was sequenced to ensure its authenticity. For the luciferase assay, 293 T cells were transfected with 0.2 mg luciferase reporter constructs as described above at the same time with miR-125b-5p mimic (100 nM) or control RNAs using Lipofectamine 2000 (Invitrogen). After 48 hr, a luminometer (Promega) was used to determine the luciferase activity under a dual-luciferase reporter assay system (Promega).

### 2.14. Statistical Analyses

The measurement data are presented as the mean ± standard deviation (SD). Multiple samples were compared by one-way analysis of variance to determine whether there were significant differences. The comparison of each group was performed by an LSD *t*-test, and the Dunnett *t*-test was used to compare the ELISA results. *P* < 0.05 was considered statistically significant. All data were analyzed by SPSS 19.0 software (IBM Corp. USA).

## 3. Result

### 3.1. Isolation and Characterization of iMSC-sEV

**Figure 1:**
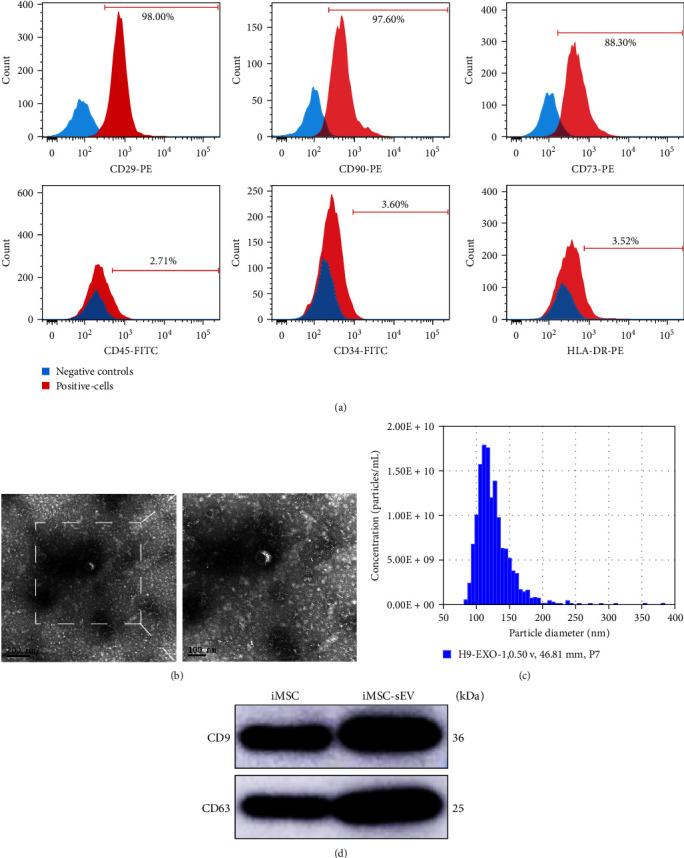
Characterization of induced pluripotent stem cell-derived mesenchymal stem cells and iMSC-sEV. (a) Flow cytometric analysis of the surface markers in iMSCs. (b) Morphology of iMSC-sEV observed by TEM (scale bar, 200 and 100 nm). (c) Particle size distribution of iMSC-sEV measured and identified through NanoSight tracking analysis. (d) Representative western blot assessment of iMSC-sEV and iMSCs lysates showing the presence of sEV markers, including CD9 and CD63.

Flow cytometric analysis was used to detect the expression of MSC characteristic markers in these induced cells. The result demonstrated that the majority of iMSC were highly positive for CD29, CD90, and CD73 and were negative for CD45, CD34, and HLA-DR (Figure 1(a)). Thereafter, iMSC-sEV were isolated from the conditional medium of MSCs and characterized by TEM, western blotting and nanoparticle analysis. The TEM image showed that iMSC-sEV exhibited a typical cup-shaped morphology (Figure 1(b)). Western blotting analyses indicated that iMSC-sEV expressed sEV specific markers CD63 and CD9. NanoSight tracking analysis determined that iMSC-sEV had a mean diameter of approximately 130 nm. These data demonstrate that we have successfully extracted and purified iMSC-sEV from the iMSC culture medium.

### 3.2. iMSC-sEV Display Anti-Inflammatory Properties in the Rat Model of Septic Lung Injury

**Figure 2:**
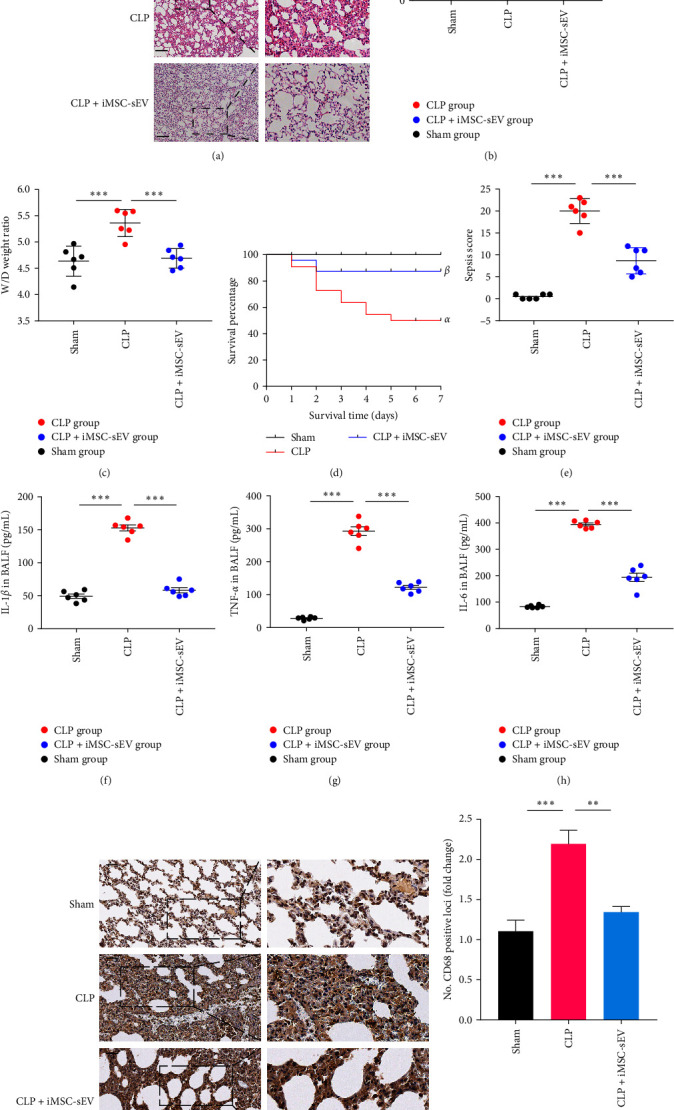
iMSC-sEV significantly alleviated septic lung injury and reduced pulmonary inflammation in rats. (a, b) Lung histopathology examination and lung injury scores as determined by H&E staining. Scale bar, 100 *μ*m. (c) The wet/dry weight ratio in lung tissue. (d) Survival rate of rats in each group within 7 days. The Kaplan–Meier method combined with the log-rank test was used to compare multiple populations. Compared with the sham group, *αP* < 0.01; compared with CLP group, *βP* < 0.01; *n* = 20 for each group. (e) The changes in sepsis score of each group. (f–h) Levels of IL-1*β*, TNF-*α*, and IL-6 in BALF were detected by ELISA. (i) Immunohistochemical for CD68 positive macrophage in the lung tissue. Scale bar, 50 *μ*m. (j) The numbers of CD68 positive loci were quantified with ImageJ software. Data presented as mean ± SD.  ^*∗∗*^*P* < 0.01,  ^*∗∗∗*^*P* < 0.001 compared within two groups. *n* = 6 per group.

Rats were subjected to CLP to construct a septic lung injury model. Twenty-four hours after the CLP treatment, typical lung injury phenotypes were observed, such as histological characteristics of lungs, an increased lung W/D weight ratio, and an increased inflammatory cytokines concentration in bronchoalveolar lavage fluid (BALF) (Figure 2(a)–2(h)). To further explored the potential role of iMSC-sEV in pulmonary inflammation and lung injury, iMSC-sEV (2 mg/kg) were intraperitoneally administered 4 hr after performing CLP in the rats. Intriguingly, we discovered that treatment with iMSC-sEV significantly reduced the pathological score of lung injury, alleviated the infiltration of inflammatory cells, and protected the integrity of the alveolar structure in lung tissue (Figure 2(a)–2(c)). As shown in Figure 2(f)–2(h), iMSC-sEV intervention inhibited the content of inflammatory factors in BALF. Moreover, iMSC-sEV improved the survival rate of septic lung injury rats and decreased the sepsis score in the lung, as compared with the CLP rat (Figures 2(d) and 2(e)). AMs, as the main immune cells in lung, play an important role in the process of acute lung injury. To examine the interaction between iMSC-sEV and AMs, we further used the immunohistochemical method of CD68-labeled AMs to observe the infiltration of macrophages in lung tissue. The CLP rat exhibited a marked increase of CD68 positive macrophages infiltration in the lungs, which was protected by iMSC-sEV treatment (Figures 2(i) and 2(j)). The data showed that iMSC-sEV display anti-inflammatory properties that protect the lungs against sepsis-induced inflammation and structural damage, which is likely related to the functional regulation of AMs.

### 3.3. iMSC-sEV Attenuates LPS-Induced Inflammatory Response in AMs

**Figure 3:**
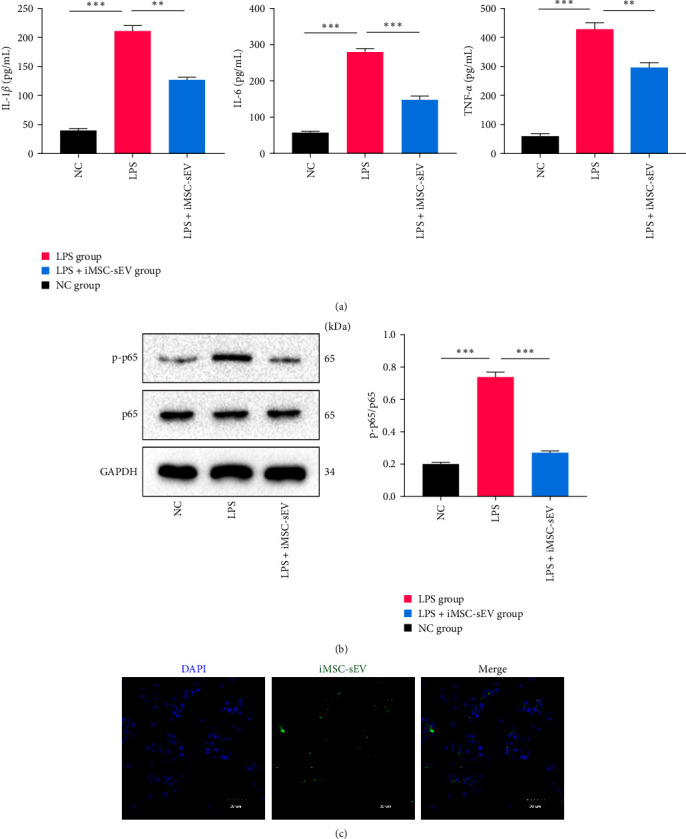
iMSC-sEV ameliorated AMs inflammatory response and inhibited the NF-*κ*B signaling pathway activation induced by LPS in vitro. (a) Pro-inflammatory cytokines IL-1*β*, TNF-*α*, and IL-6 levels in the supernatant of AMs treated with iMSC-sEV. (b) Representative western blot and quantitative analysis of p-p65 and p65 in recipient AMs treated with iMSC-sEV. (c) Detection of the uptake effects of AMs on PKH-67-labelled iMSC-sEV by means of fluorescent microscopy. Data presented as mean ± SD.  ^*∗∗*^*P* < 0.01,  ^*∗∗∗*^*P* < 0.001 compared within two groups. *n* = 3 per group.

In order to explore the anti-inflammatory effects of iMSC-sEV on AMs inflammatory response, we cocultured iMSC-sEV (50 *μ*g/mL) with LPS (50 ng/mL) prestimulated rat alveolar macrophage cell lines (NR8383) for 24 hr. As shown in Figure 3(a), LPS substantially increased the pro-inflammatory cytokines, including TNF-*α*, IL-6, and IL-1*β*. However, treatment with iMSC-sEV significantly attenuated these increases. Similarly, this change was also accompanied by reduced expression of the LPS-induced p-p65 protein (Figure 3(b)). To assess the colocalization of iMSC-sEV with AMs, we labeled iMSC-sEV with PKH-67. As shown in Figure 3(c), green fluorescently labeled iMSC-sEV surround the nucleus of AMs, suggesting that iMSC-sEV entered into LPS-treated NR8383 cells. Taken together, these results indicate that a potential role of iMSC-sEV in alleviating AMs is to release inflammatory factors by inactivating the NF-*κ*B signaling pathway.

### 3.4. miRNAs Profile of LPS-Treated NR8383 Cells under iMSC-sEV Coculture

**Figure 4:**
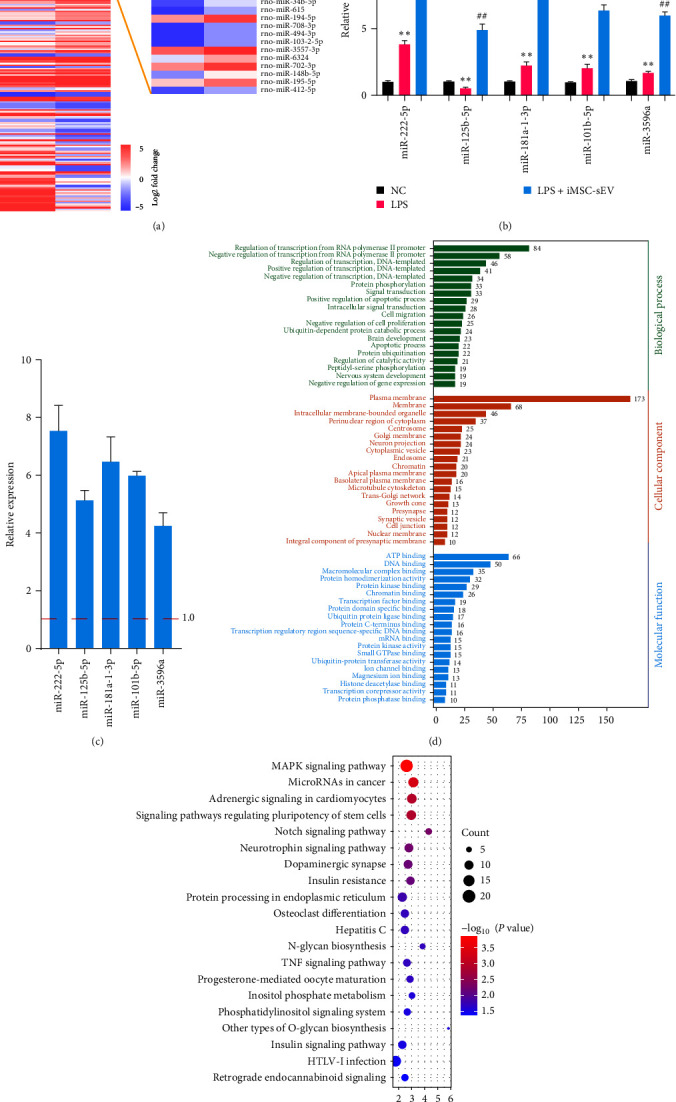
Small RNA sequencing for analysis of miRNAs in LPS-treated NR8383 cells under iMSC-sEV coculture. (a) Heatmap showing the differential expression level of miRNAs between LPS-treated NR8383 cells under iMSC-sEV coculture or not. (b, c) The RT-qPCR analysis validated the top five upregulated miRNAs in LPS-treated NR8383 cells and iMSC-sEV, in which miR-125b-5p was identified as one of the significant upregulated miRNAs in iMSC-sEV. (d, e) The Kyoto Encyclopedia of Genes and Genomes (KEGG) analysis and gene ontology (GO) were performed on the predicted target genes of miR-125b-5p. Data presented as mean ± SD compared with the NC group,  ^*∗∗*^*P* < 0.01; compared with LPS group, ##*P* < 0.01. *n* = 3 per group.

It was reported that miRNAs encapsulated in sEV are important mediators of information exchange between cells. To further identify the key miRNAs that contributed to the anti-inflammatory effect of iMSC-sEV, we performed total small RNA sequencing to detect the miRNA profile in LPS-treated NR8383 cells under iMSC-sEV coculture. LPS-treated NR8383 cells were used as controls. As a result, a total of 160 miRNAs were differentially expressed between LPS-treated NR8383 cells and LPS-treated NR8383 cells under iMSC-sEV coculture (minimum fold change of two and adjusted *P*-value < 0.05), among which 88 of miRNAs were higher in the LPS + iMSC-sEV group (Figure 4(a)). The top five upregulated miRNAs in NR8383 cells were validated by qRT-PCR, and their expression levels in iMSC-sEV were also detected (Figures 4(b) and 4(c)). Notably, we found that LPS treatment markedly decreased the expression of miR-125b-5p in NR8383 cells compared with normal cells, while the higher level of miR-125b-5p was detected in LPS-treated NR8383 cells under the induction of iMSC-sEV, which indicated iMSC-sEV may transfer miR-125b-5p to AMs. In addition, the expression level of miR-125b-5p showed that it was highly enriched in iMSC-sEV, and this molecule has previously been reported to ameliorate inflammation. Subsequently, the target genes of miR-125b-5p, predicted by the TargetScan database, were subjected to a bioinformatics enrichment analysis. As shown in Figures 4(d) and 4(e), GO and the KEGG analysis revealed that the genes targeted by miR-125b-5p were significantly involved in the immune- and inflammation-related pathways, such as the MAPK, Notch, and TNF-*α* signaling pathway. These results indicated that iMSC-sEV contain miR-125b-5p, which might serve as a potential candidate mediating the anti-inflammatory effects of iMSC-sEV in the sepsis-induced acute lung injury.

### 3.5. MiR-125b-5p-Mediated Anti-Inflammatory Effects of iMSC-sEV on Septic Lung Injury

**Figure 5:**
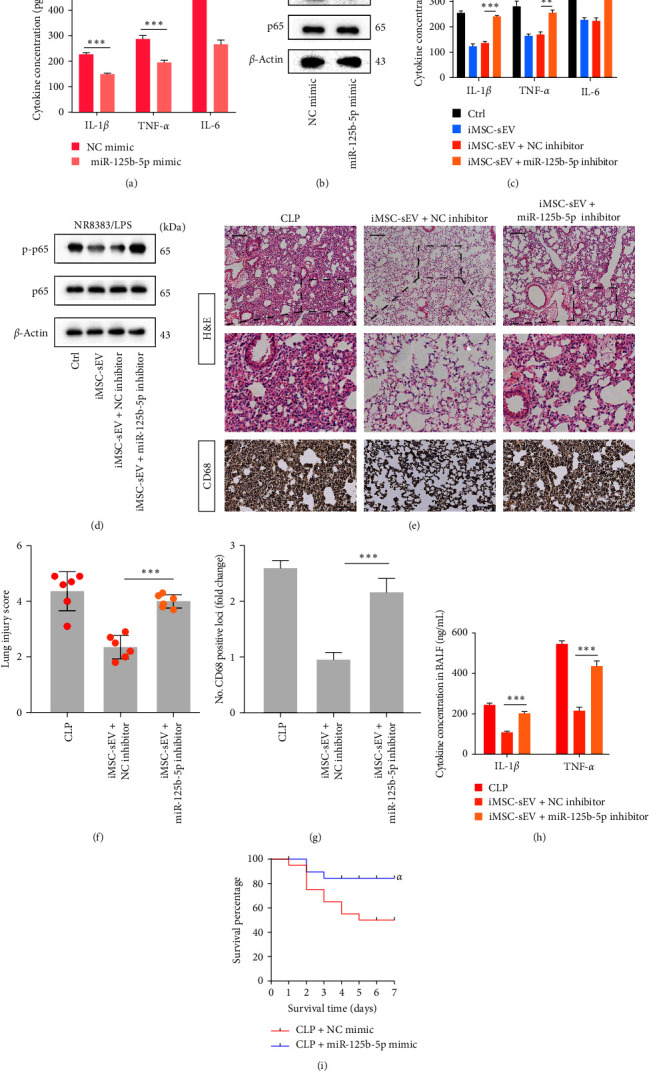
Anti-inflammatory effects of iMSC-sEV on AMs were mediated by miR-125b-5p in vitro and in vivo. (a, b) ELISA examined the level of inflammatory cytokine expression and NF-*κ*B signaling pathway in LPS-treated NR8383 cells transfected with miR-125b-5p mimics. (c, d) Detection of inflammatory cytokines and NF-*κ*B signaling pathway in LPS-treated NR8383 cells with the iMSC-sEV or iMSC-sEV +miR-125b-5p inhibitor. (e) Changes in histologic (H&E staining) and CD68 positive macrophage infiltration in the lung tissue. (f) Lung injury score of rats in each group. (g) The numbers of CD68 positive loci were quantified with ImageJ software. (h) The ELISA analysis showed the concentration of intrapulmonary inflammatory cytokines (IL-1*β*, TNF-*α*) in the BALF. (i) Survival rate of rats in each group within 7 days. The Kaplan–Meier method combined with the log-rank test was used to compare multiple populations. Compared with the CLP + NC mimic group, *αP* < 0.01; *n* = 20 for each group. Data presented as mean ± SD.  ^*∗∗∗*^*P* < 0.001,  ^*∗∗*^*P* < 0.01 compared within two groups. *n* = 6 per group.

To further determine the biological role of miR-125b-5p in LPS-treated NR8383 cells, overexpression of miR-125b-5p with specific miRNA mimics was transfected into NR8383 cells. The level of proinflammatory cytokines such as IL-1*β*, TNF-*α*, and IL-6 and p65 phosphorylation were both significantly decreased after overexpressing miR-125b-5p in the LPS-treated NR8383 cells (Figures 5(a) and 5(b)). In contrast, as shown in Figure 5(c), inhibition of miR-125b-5p partly recovered the level of proinflammatory cytokines weakened by iMSC-sEV. Meanwhile, the western blot analysis also revealed that the level of p-p65 was alleviated with the treatment of iMSC-sEV, while the tendency was partly reversed after cocultured with the miR-125b-5p inhibitor (Figure 5(d)). Furthermore, we evaluated the effect of miR-125b-5p in iMSC-sEV in a rat model of septic lung injury. Similar to findings in vitro, we found that the miR-125b-5p inhibitor had weaker effects on the reduction of CD68+ alveolar macrophage infiltration and modest lung injury compared with the iMSC-sEV + NC inhibitor (Figure 5(e)–5(g)). Accordingly, the miR-125b-5p inhibitor markedly increased the expression of lung inflammatory cytokines (Figure 5(h)). More importantly, as with the role played by iMSC-sEV, miR-125b-5p mimics were intratracheally administered into the sepsis rats also improved the survival rate of septic rats (Figure 5(i)). Totally, iMSC-sEV inactivated AMs and attenuated pulmonary inflammation through a miR-125b-5p-dependent pathway.

### 3.6. iMSC-sEV-Shutting miR-125b-5p Suppresses NF-*κ*B Signaling in Macrophages by Targeting TRAF6

**Figure 6:**
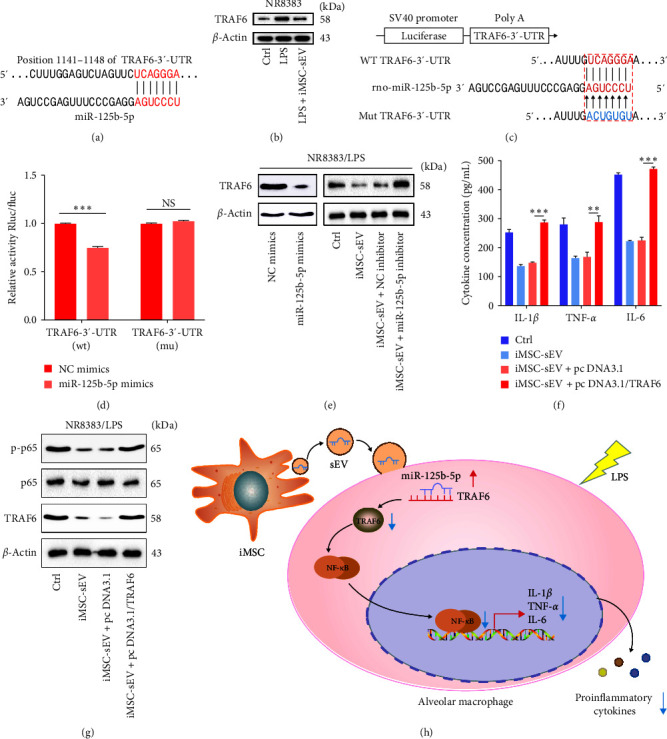
miR-125b-5p transmitted by iMSC-sEV suppresses NF-*κ*B signaling in alveolar macrophages by directly targeting TRAF6. (a) Online TargetScan v7.2 predicted miR-125b-5p targeted to TRAF6. (b) Protein expression of TRAF6 in LPS-treated NR8383 cells under iMSC-sEV coculture. (c, d) The luciferase reporter assay examined the luciferase activity of the indicated reporter vectors in 293 T cells cotransfected with miR-125b-5p mimics or NC mimics. (e) Western blot analysis detected the protein levels of TRAF6 after transfection with the miR-125b-5p mimic or miR-125b-5p inhibitor. *β*-Actin was used as an internal control. (f) ELISA examined the level of inflammatory cytokine expression in LPS-treated NR8383 cells when overexpressed with TRAF6. (g) The western blot analyzed the protein level of p-p65 in LPS-treated NR8383 cells when overexpressed with TRAF6. Rescue of TRAF6 expression dramatically inhibited iMSC-sEV to inactivate p65 phosphorylation. *β*-Actin was used as an internal control. (h) The concept map demonstrating the role and functional mechanism of iMSC-sEV on alleviating septic lung injury. Data presented as mean ± SD.  ^*∗∗∗*^*P* < 0.001 compared within two groups. NS, no significant difference, *n* = 3 per group.

Next, the putative mechanism responsible for iMSC-sEV transferred miR-125b-5p-mediated AMs function and suppression of NF-*κ*B signaling was elucidated. The systematic bioinformatics analysis indicated that a miR-125b-5p binding site was identified in the 3ʹ-UTR of TRAF6 (Figure 6(a)). TRAF6 has been shown to play an important role in NF-*κ*B activation. Therefore, the western blot analysis of TRAF6 expression was performed on NR8383 cells. As expected, TRAF6 expression was significantly repressed in miR-125b-5p-enriched iMSC-sEV treated NR8383 cells (Figure 6(b)). Furthermore, a dual luciferase assay was performed with WT and mutant (MUT) constructs of the TRAF6 3ʹ-UTR to assess the interaction between TRAF6 and miR-125b-5p. The results showed that the miR-125b-5p mimic significantly decreased the luciferase reporter activity of the WT-TRAF6 plasmid by approximately 30%. Conversely, these effects were abolished when the MUT 3ʹ-UTR construct was used, indicating a specific interaction between miR-125b-5p and the 3ʹ-UTR of TRAF6 (Figures 6(c) and 6(d)). Meanwhile, the western blot analysis also revealed that the level of TRAF6 in LPS-treated cells was reduced after overexpressing miR-125b-5p. Interestingly, we disclosed that the decreased level of TRAF6 protein caused by iMSC-sEV was partly recovered by the miR-125b-5p inhibitor (Figure 6(e)). Based on the aforementioned results, we further explored whether the effects elicited by transferred iMSC-sEV could be reversed by increasing the TRAF6 levels. As shown in Figures 6(f) and 6(g), decreased levels of proinflammatory cytokines and phosphorylated NF-*κ*B induced by iMSC-sEV were increased due to TRAF6 overexpression. All these results suggested that iMSC-sEV-shutting miR-125b-5p suppresses NF-*κ*B signaling in macrophages by targeting TRAF6.

## 4. Discussion

Septic lung injury is a devastating respiratory disorder, which leads to mortality in patients in intensive care units [6]. The treatment still remains primarily supportive, and there is a lack of effective approaches targeting the life-threatening illness to date. Increasing studies have shown that AMs are key factors in the pathogenesis of septic lung injury by releasing excessive and uncontrolled inflammatory responses [26, 27]. In the current study, we found that iMSC-sEV were able to modulate AMs function in vitro and attenuate sepsis pulmonary inflammation in an experimental rat model, at least in part due to the transfer of miR-125b-5p to AMs (Figure 6(h)). Our findings further demonstrated that iMSC-sEV could serve as a novel and promising cell-free agents for the treatment of septic lung injury.

In recent years, the implantation of stem cells has achieved very gratifying application prospects for the treatment of immune diseases and regenerative medicine. MSCs are considered to be the gold standard of stem cells for autologous transplantation therapy. The therapeutic effects on lung injury and lung disease in MSC from various sources such as bone marrow, adipose tissue, and human umbilical [28–30]. However, the current application of MSCs still faces several challenges, as the number of MSCs obtained from donors is often insufficient. In addition, the in vitro growth and differentiation potential is affected by various factors such as the age of the donor, the health condition of the donor, and the culture period. By contrast, iMSCs from iPSCs possess desirable advantages; not only do they overcome the limitation of traditional MSCs, but they also avoid ethical problems and immune rejection, providing an ideal approach for the large-scale application of MSCs. In the present study, we used the modified one-step induction protocol to induce the differentiation of iPSCs into iMSCs. The detection of cell surface markers such as CD29, CD90, and CD73 by flow cytometry confirmed that they were in line with the characteristics of MSCs.

It has been suggested that the main potential mechanism of stem cell transplantation therapy depends on the paracrine activity. Among these paracrine bioactive substances, sEV secreted by cells are attracting increasing interest. MSC-sEV have become promising immunomodulators as they possess similar properties to their parent MSCs [31–33]. Emerging studies have investigated the therapeutic effects of MSC-sEV against sepsis-induced acute lung injury, in which MSC-sEV ameliorated lung pathological damage and significantly reduced inflammation infiltration [34, 35]. However, to the best of our knowledge, the role of iMSC-sEV in the context of sepsis-induced acute lung injury and the underlying mechanisms are largely unclear. In this study, our results showed that intraperitoneally administered with iMSC-sEV remarkably ameliorated interstitial edema, reduced CD68 positive AMs migration, and improved the survival of CLP-induced septic lung injury rats in the model, confirming the ability of iMSC-sEV to mitigate airway inflammation and reduce lung injury under sepsis conditions. More importantly, through tracking sEV with PKH-67, we observed that iMSC-sEV were internalized by AMs and significantly decreased the level of pro-inflammatory cytokines in vitro. The effects of iMSC-sEV on AMs were evidenced by the inactivation of the NF-*κ*B signaling pathway. In light of AMs are central mediators in the development of sepsis-induced acute lung injury, contributing to the initiation of inflammatory response [36, 37]. We propose that iMSC-sEV exerts an anti-inflammatory effect against septic lung injury may benefit from the interaction between iMSC-sEV and AMs.

sEV are rich in a variety of miRNAs, one of the most important active components for their biological functions. The miRNAs encapsulated in sEV are transferred to recipient cells, regulate the expression of downstream target genes post-transcriptionally, and largely decide the effects of sEV on recipient cells [38]. Accumulated evidence indicates that miRNAs in EVs derived from stem cell possess immunomodulatory properties [39–41]. Presently, we provide differential expression profiles of miRNAs in the LPS-treated NR8383 cells under iMSC-sEV cocultured. In particular, among these upregulated miRNAs, miR-125b-5p is highly conservative among species and abundant in iMSC-sEV but is expressed at low levels in LPS-treated AMs. miR-125b-5p has been reported to regulate inflammation response and protect from sepsis-induced acute lung injury [42]. Furthermore, miR-125b-5p was inhibited in nonsurvivors with sepsis and was downregulated in BALF from LPS-induced acute lung injury mice [43, 44]. Inhibition of miR-125b-5p could impair the suppressive action of metformin on pro-inflammatory factor production [45]. This evidence, along with our present findings, showed that miR-125b-5p might play a vital role in iMSC-sEV regulating alveolar macrophage inflammation.

The exact mechanism by which miR-125b-5p regulates macrophage activation requires further investigation. When AMs are stimulated by microorganisms, NF-*κ*B signaling induces AMs to secrete a variety of inflammatory transmitters and aggravates lung injury [46]. Among the possible candidates, mRNA targets of miR-125b-5p were predicted by TargetScan assays. TRAF6 is closely associated with the activation of the NF-*κ*B signaling pathway and plays an important role in the process of acute lung injury and inflammation [47, 48]. A dual-luciferase assay was performed to confirm that miR-125b-5p can bind to the predicted binding site in the TRAF6 3ʹ-UTR. This study found that TRAF6 is a target gene of miR-125b-5p. After treatment with iMSC-sEV enriched with miR-125b-5p showed a significant decrease in TRAF6 expression in LPS-treated AMs, parallel with the suppression of NF-*κ*B and pro-inflammatory cytokines.

This study does have several limitations: (1) Different concentrations of iMSC-sEV should be investigated in dose/response experiments, which are used to evaluate the dosage of clinical medication. (2) Whether other substances in iMSC-sEV, such as proteins, mRNA, and lipids, in iMSC-sEV-mediated lung protection in septic lung injury remains to be investigated in future studies.

Taken together, the present study reported that iMSC-sEV protects against septic lung injury and exerts anti-inflammatory effects on AMs. We demonstrated that iMSC-sEV transmit miR-125b-5p into LPS-treated NR8383 cells to target TRAF6, thereby inactivating the NF-*κ*B signaling pathway. Furthermore, our findings suggested that iMSC-sEV may provide a novel cell-free utility value for the treatment of septic lung injury.

## Data Availability

All data generated or analyzed in this study are included in the published article.

## Abbreviations

CLP: Cecal ligation and puncture
iPSC: Induced pluripotent stem cell
MSCs: Mesenchymal stem cells
iMSCs: Induced pluripotent stem cell-derived mesenchymal stem cells
iMSC-sEV: Small extracellular vesicles derived from iMSCs
BALF: Bronchoalveolar lavage fluid
AMs: Alveolar macrophages
TRAF-6: TNF receptor-associated factor 6
NF-*κ*B: Nuclear factor-*κ*B
EVs: Extracellular vesicles
sEVs: Small extracellular vesicles
IL-1*β*: Interleukin-1*β*
TNF-*α*: Tumor necrosis factor *α*.
